# Effects of Adding Ultrasound-Guided Percutaneous Neuromodulation to a Percutaneous Needle Electrolysis and Eccentric Exercise Intervention in Patellar Tendinopathy: A Randomized Controlled Trial

**DOI:** 10.3390/healthcare14142092

**Published:** 2026-07-13

**Authors:** Ignacio Molina, Fermín Valera-Garrido, Francisco Minaya-Muñoz, José Ignacio Molina, Pablo Herrero, Diego Lapuente-Hernández, Alberto Carcasona-Otal

**Affiliations:** 1 JIM Fisioterapia S.L., 28016 Madrid, Spain; nachomolina1999@gmail.com (I.M.); jimfisioterapia@yahoo.es (J.I.M.); 2 MVClinic Institute, 28600 Madrid, Spain; ferminvalera@mvclinic.es (F.V.-G.); franminaya@mvclinic.es (F.M.-M.); 3 Faculty of Medicine, CEU San Pablo University, 28925 Madrid, Spain; 4 Invasive Physiotherapy Department, Getafe Football Club, 28903 Madrid, Spain; 5 Department of Physiatry and Nursing, Faculty of Health Sciences, University of Zaragoza, 50009 Zaragoza, Spain; pherrero@unizar.es (P.H.); acarcasona@unizar.es (A.C.-O.); 6 iHealthy Research Group, Institute for Health Research Aragón, Hospital Clínico Universitario Lozano Blesa, 50009 Zaragoza, Spain

**Keywords:** patellar tendon, exercise, invasive physiotherapy, percutaneous needle electrolysis, percutaneous electrical nerve stimulation, ultrasonography

## Abstract

**Highlights:**

**What are the main findings?**

- The addition of ultrasound-guided percutaneous neuromodulation to an intervention involving percutaneous needle electrolysis and eccentric exercise did not demonstrate any statistically significant additional benefits in terms of pain intensity, function, quality of life or ultrasound imaging findings.
- Both groups demonstrated clinical and structural improvements, highlighting important reductions in hypoechoic areas within the tendon, neovascularization, and tendon thickening, with no cases of deterioration observed.

**What are the implications of the main findings?**

- Progressive loading through exercise and the selective application of ultrasound-guided percutaneous needle electrolysis are key therapeutic components for patellar tendinopathy, suggesting the hypothesis of a potential therapeutic ceiling effect that limits the added value of other complementary invasive techniques, though this requires further investigation.
- Ultrasound-guided percutaneous neuromodulation may play a role in achieving clinically relevant improvements in certain outcomes, but its added value must be assessed on a case-by-case basis, and requires further research.

**Abstract:**

**Background/Objectives**: Eccentric exercise (EE) combined with percutaneous needle electrolysis (PNE) is an evidence-based treatment for patellar tendinopathy (PT). Percutaneous neuromodulation (PNM) is a promising adjunctive technique, yet its complementary effect in PT remains unstudied. This study aimed to determine whether adding ultrasound-guided PNM to an intervention based on PNE and EE provides additional benefits regarding pain intensity, function, quality of life and ultrasound imaging variables in individuals with PT. **Methods**: A single-blinded randomized clinical trial was conducted with 26 participants with PT, allocated to an active control group (EE+PNE) or an experimental group (EE+PNE+PNM). Percutaneous interventions were applied on days 1, 7, and 14, while EE was performed twice daily for four weeks. Pain intensity (VAS) was assessed at days 1, 7, 14, and 21, and analyzed using a linear mixed-effect model. Remaining outcomes (VISA-P, SF-36, pistol squat and ultrasound imaging) were assessed at days 1 and 21 and analyzed accordingly using either ANCOVA or non-parametric methods. **Results**: Significant reductions in pain intensity were observed over time in both groups (*p* < 0.001), with no between-group differences (*p* = 0.844) or group x time interaction (*p* = 0.561). No significant between-group differences were found for VISA-P (*p* = 0.181), SF-36 (*p* = 0.400), functional capacity (*p* = 0.716), or ultrasound imaging outcomes (*p* > 0.05 for all of them). **Conclusions**: Adding ultrasound-guided PNM to an EE+PNE protocol did not provide statistically significant short-term additional benefits over EE+PNE alone in individuals with PT. Further research with larger samples and longer follow-up is warranted.

## 1. Introduction

Patellar tendinopathy (PT), also known as “jumper’s knee,” is characterized by degeneration of the tendon, primarily due to excessive structural overuse. This leads to a progressive increase in anterior knee pain and dysfunction of the affected tendon [1]. The condition also causes various changes within the tissue, including tendon thickening, collagen fiber degeneration and disorganization, fibroblast proliferation with incomplete repair of injury, neovascularization, and neurogenesis [2,3]. Several studies report a prevalence of PT of 18.3% among athletes and 0.1% among the general population [4]. A prospective follow-up study found that over 50% of athletes with PT had to withdraw from competitive sports [5]. Males over 18 years old who engage in sports such as volleyball, basketball, or soccer are more commonly affected [4].

Various diagnostic methods can be employed in both medical [2,6] and physiotherapy [7,8,9] settings to identify PT. Clinical symptoms include anterior knee pain that worsens with exercise, after exercise, or with prolonged knee flexion, as well as localized pain and hypersensitivity at the proximal tendon insertion and loss of function in movements such as squatting with ankle plantar flexion [4,8]. Imaging evaluations such as ultrasound, magnetic resonance imaging, or X-rays are also used to identify focal hypoechoic areas, neovascularization, tendon thickening, and changes to the cortical bone of the patellar inferior pole or tibial plateau [8,9].

In the advanced stages of tendon degeneration, surgical intervention may be considered, either through open procedures or arthroscopic techniques [1]. Non-surgical treatment options for PT include medical approaches such as platelet-rich plasma injections, hyaluronic acid, non-steroidal anti-inflammatory drugs, and corticosteroids [10]. However, conservative physiotherapy is generally regarded as the primary intervention, incorporating key evidence-based components for optimal recovery. These primarily comprise load management strategies involving progressive tendon loading through therapeutic exercise, particularly eccentric exercise (EE) [6,11]. Additional adjunctive interventions include extracorporeal shockwave therapy [3], manual therapy [8], functional taping [9] and cryotherapy [9].

Alongside these conservative physiotherapy interventions, invasive physiotherapy techniques such as dry needling [12,13,14] and percutaneous needle electrolysis (PNE) [14,15,16,17] have also gained relevance in the management of PT, as evidenced by recent research. Valera-Garrido et al. (2017) defined PNE as “an invasive physiotherapy technique involving the application of a galvanic current through a puncture needle under ultrasound guidance, producing an analgesic effect and a local inflammatory process in musculoskeletal soft tissue that promotes phagocytosis and tissue repair” [17]. Using the needle directly on the tendon induces histological changes in the affected tissue.

Another technique used in invasive physiotherapy is percutaneous neuromodulation (PNM), which has a longer history and a greater body of evidence, with the first study conducted by Campbell and Taub in 1973 [18]. PNM involves the electrical stimulation of a peripheral nerve or a muscle at a motor point for therapeutic purposes using a needle guided by ultrasound [17]. Applying this treatment with appropriate parameters (frequency, intensity, application time, etc.) can induce changes in the patient’s somatosensory, somatomotor, and autonomic nervous systems [18]. This is particularly useful in cases where one or more of these systems are affected by the type, severity, or chronicity of the pathology [18,19].

Within the current literature, load management strategies (mainly through EE) constitute the cornerstone of recovery in PT [11]. Furthermore, adding PNE to these load management strategies has been shown to be an effective treatment for tendinopathies [12,15,16,20]. However, evidence regarding the use of PNM as a complementary intervention to the combined use of load management strategies (e.g., EE) and PNE in tendinopathies remains very limited. We hypothesize that it could be a valuable tool for modulating symptoms, particularly in persistent conditions involving chronic pain, and for improving neuromuscular function, which tends to decline progressively in this population due to tendon degeneration [21].

Therefore, a randomized controlled trial was proposed to analyze whether adding a specific ultrasound-guided PNM treatment to the intervention consisting of EE+PNE provides additional benefits in terms of pain reduction, functional improvement, and quality of life in individuals with PT. In addition, structural changes assessed through ultrasound imaging were evaluated to support future descriptive analysis of these ultrasound findings.

## 2. Materials and Methods

### 2.1. Study Design

A single-blinded randomized clinical trial was conducted, in which only the evaluator was unaware of group allocation. Participants were recruited in Madrid, Spain, between May and August 2025. All assessments and interventions were conducted at JIM FISIOTERAPIA S.L. The total sample was divided into two intervention groups: an active control group that received ultrasound-guided PNE alongside an EE protocol (EE+PNE), and an experimental group that received the same intervention with the addition of ultrasound-guided PNM targeting the femoral nerve (EE+PNE+PNM). The randomization sequence for allocating treatments was generated using the random sampling function in IBM SPSS Statistics (Statistical Package for the Social Sciences, version 30.0.0.0.). Then, to ensure allocation concealment, the sequence was placed in sequentially numbered, opaque, sealed envelopes by an independent researcher not involved in the assessment or treatment of the participants. The blinded evaluator (I.M.) was responsible for enrolling participants and conducting all baseline and follow-up assessments. Following the baseline assessment, the unblinded physical therapist (J.I.M.) opened the corresponding envelope to assign the participant to their allocated intervention.

This study was conducted in accordance with the 2025 CONSORT guidelines [22] (see Supplementary Materials), the 2013 Declaration of Helsinki of the World Medical Association [23], and Spanish Organic Law 3/2018 on the Protection of Personal Data and Guarantee of Digital Rights. These guidelines ensured anonymity and confidentiality. This study also complied with the provisions of Law 14/2007 on Biomedical Research Involving Human Subjects. Approval was obtained from the Ethics Committee of Aragon (Reference: C.I. PI24/528, dated 15 January 2025), and this study was registered with ClinicalTrials.gov (NCT06685302).

### 2.2. Participants

Participant recruitment started in May 2025 in Madrid, Spain. The researchers sought collaboration from fellow healthcare professionals, their personal networks, public information-sharing groups, and local sports centers, gyms and athletic clubs. A digital questionnaire was used to pre-screen potential participants and establish initial contact. This questionnaire was used to screen out conditions unrelated to the patellar tendon, such as involvement of the medial and lateral collateral ligaments of the knee, meniscal pathologies, and quadriceps tendon disorders, as well as the prolonged use of anti-inflammatory drugs and infiltration-based treatments.

Participants were eligible for inclusion if they met the following criteria: (1) aged between 18 and 50 years, in accordance with previous systematic reviews and meta-analyses which reported a higher prevalence of patellar tendinopathy (PT) within this age range [4,24,25]; (2) both male and female participants; and (3) a clinical diagnosis of PT based on the following: (a) anterior knee pain localized to the inferior pole of the patella for at least two weeks; (b) a score of less than 80 points on the Spanish version of the Victorian Institute of Sport Assessment–Patella (VISA-P) questionnaire; and (c) ultrasonographic findings consistent with PT in both the transverse and longitudinal planes of the patellar tendon. These findings included: focal hypoechoic areas; focal hyperechoic areas; tendon thickening compared with the contralateral side; loss of continuity of the deep interface between the patellar tendon and Hoffa’s fat pad; and the presence of intratendinous neovascularization [17]; (4) physically active individuals, defined as engaging in at least 150 min of moderate-intensity physical activity, or 75 min of vigorous-intensity physical activity, or some equivalent combination of both per week, in accordance with the minimum recommendations of the World Health Organization 2020 Guidelines [26]; (5) provision of written informed consent following a thorough explanation of the study procedures.

Participants were excluded if they met any of the following criteria: (1) presence of any pathology or severe chronic condition affecting the lower limbs (e.g., Osgood–Schlatter disease or Sinding–Larsen–Johansson syndrome); (2) use of any pharmacological treatment or intervention of physiotherapy targeting the knee within 48 h prior to the intervention; (3) history of knee surgery within the previous year; (4) receipt of corticosteroid injections in the knee within three months prior to the intervention; (5) presence of any contraindication to receiving invasive techniques (e.g., belonephobia, allergy to materials used, oncological conditions, thrombophlebitis, dermatological disorders, fever, etc.).

### 2.3. Sample Size Calculation

A sample size calculation was performed to detect differences in pain intensity over time between groups, using a linear mixed-effect model with pain, as measured by the Visual Analog Scale (VAS), as the primary outcome. Based on previous studies with similar populations, a standard deviation of 0.7 points on this scale was assumed [13], and a minimal clinically important difference of 2 points was identified. The model included fixed effects for treatment group, time (Day 7, Day 14, and Day 21), and their interaction. Baseline VAS (Day 1) was included as a covariate. As there are currently no specific values for the within-subject correlation (intraclass correlation coefficient, ICC) for VAS in PT, values reported in related knee pain populations were considered. Previous studies have reported high ICC values for VAS, including 0.97 for osteoarthritic knee pain [27]. Based on this evidence, a very conservative ICC value of 0.7 was assumed for the present calculation. A significance level of 5% (α = 0.05) and a statistical power of 90% (1 − β = 0.90) were established. The estimated required sample size was 24 participants. To account for potential dropouts, the final sample size was increased to 26 participants, with 13 per group.

### 2.4. Outcomes and Assessment Points

#### 2.4.1. Baseline Assessment (Day 1)

Participants were examined by an experienced physical therapist who was unaware of the treatment being administered. Before the treatment was applied and the informed consent form was signed, the study protocol was explained to all participants. Any questions they had were addressed in detail, and they were reminded that they could withdraw from this study at any time without consequence.

Firstly, an evaluation was performed to confirm the PT diagnosis based on the inclusion criteria, including ultrasound assessment. For this evaluation, participants were positioned supine with their knee flexed to 30° and supported by a small pillow. The patellar tendon was examined using a diagnostic ultrasound system (ESAOTE MyLabSeven eHD, Esaote, Genoa, Italy) with a linear transducer (ESAOTE SL1543, Esaote, Genoa, Italy). Both longitudinal and transverse views were taken to identify the affected region of the tendon, which was classified according to the MVClinic Institute protocol for lesion mapping [17] as follows: (A1) proximal insertion of the deep fibers of the patellar tendon at the patella; (A2) deep interface between the patellar tendon and Hoffa’s fat pad; (A3) tendon body; (A4) distal insertion of the deep fibers of the patellar tendon at the tibia; and (A5) distal insertion of the superficial fibers of the patellar tendon at the tibia.

Before starting the intervention, the following sociodemographic outcomes were collected: age (in years); dominant side (left or right); affected limb (left or right); height (in cm); weight (in kg); and chronicity of the condition (three months to one year; one to three years; or more than three years).

Clinical outcomes were also collected at baseline, concretely pain intensity (primary outcome), which was evaluated using the Visual Analog Scale (VAS), where 0 indicates ‘no pain’ and 10 indicates ‘maximum imaginable pain’ [27]. Secondary outcomes included functionality, which was assessed using two complementary approaches. On the one hand, functional disability was measured using the VISA-P questionnaire, in which a score of 100 represents full function and the absence of symptoms. On the other hand, functional capacity was evaluated based on the ability to perform a pistol squat (single-leg squat), focusing on pain perception and scored as follows: 0 = no pain, 1 = mild pain, 2 = severe pain, and 3 = inability to perform the movement [28,29]. Quality of life was also assessed as a secondary outcome using the Spanish version of the SF-36 Health Survey, which is a 36-item, patient-reported questionnaire designed to measure general health-related quality of life across physical and mental health domains [16,30].

Quantitative outcomes were complemented with qualitative assessment through the following ultrasound outcomes: (1) focal hypoechoic areas: absent, small (<0.4 mm), or large (>0.4 mm); (2) focal hyperechoic areas: absent, small (<0.4 mm), or large (>0.4 mm); (3) tendon thickening compared with the contralateral side: absent, small (difference <1 mm), or large (difference >1 mm); (4) intratendinous neovascularization: absent, small (distance between the main vessel trunk and the most distal branch <0.5 mm), or large (distance between the main vessel trunk and the most distal branch >0.5 mm) and (5) continuity of the deep interface between the patellar tendon and Hoffa’s fat pad: preserved or not preserved. The use of these categorical classifications was chosen because this methodology has been employed in previous studies evaluating the same pathology [31,32]. Furthermore, the specific number of categories and the precise threshold limits for each ultrasound variable were established by consensus of the research team, based on the expert criteria and following previously described clinical protocols [17]. These ultrasound assessments have shown good-to-excellent inter- and intra-tester reliability, and a minimum measurement error [32,33,34,35,36].

#### 2.4.2. Follow-Up Assessment (Days 7, 14, and 21)

Participants received a second (day 7) and a third (day 14) treatment session of their allocated percutaneous intervention. The patient’s pain intensity was reassessed using the VAS before each intervention (days 7 and 14). After the third treatment session (day 14), a fourth follow-up session was held 7 days later (day 21). All outcomes were then reassessed using the same methodology as at baseline (day 1).

### 2.5. Interventions

Participants were allocated to an active control group (EE+PNE) and an experimental group (EE+PNE+PNM). Both groups performed the EE protocol and received three sessions (on days 1, 7, and 14) of ultrasound-guided PNE. The experimental group also received three sessions (on days 1, 7 and 14) of ultrasound-guided PNM. The PNE and PNM interventions employed the aforementioned ultrasound and linear transducer, as well as a medically certified device (Physio Invasiva^®^ 2.0, PRIM Physio, Madrid, Spain, Medical Devices Regulation EU 2017/745 and the European Directive 2011/65). Participants were treated by a physical therapist with over 10 years of experience in ultrasound evaluation and interventional techniques (J.I.M.). In both groups, the intervention was discontinued if serious adverse effects occurred or if the participant chose to withdraw from this study.

#### 2.5.1. Eccentric Exercise (EE) Protocol

**Figure 1:**
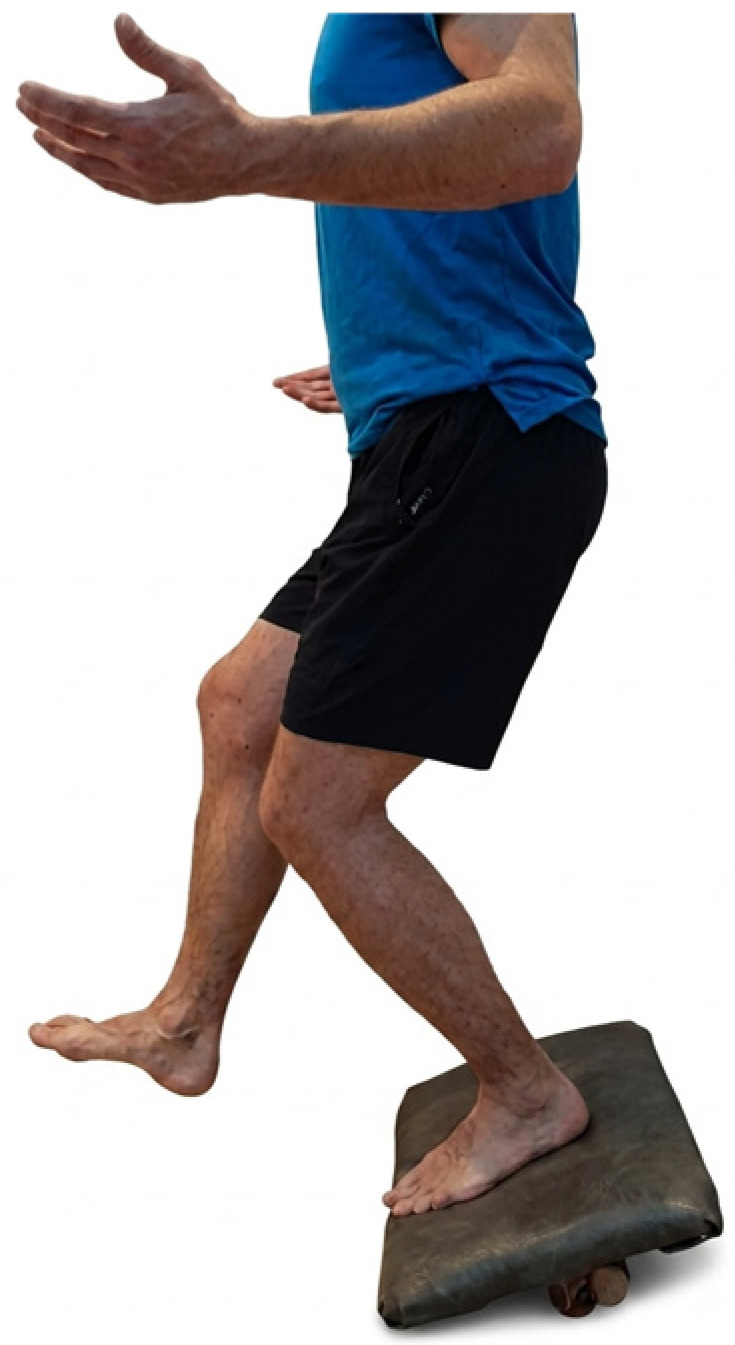
Patient performing a single-leg squat on a 25-degree decline board.

On the day of the first intervention session, each patient was instructed in performing the EE protocol. This consisted of three sets of fifteen repetitions of single-leg squats on a 25-degree decline board, performed twice daily over the four-week follow-up period (see Figure 1). Twenty-four hours of rest were allowed following each PNE or PNM intervention. This protocol was based on the approach for the treatment of PT by Young et al. [37]. Patients were also informed that they could increase the speed of execution over time, provided that any pain experienced during exercise did not exceed a VAS score of 2–3.

#### 2.5.2. Ultrasound-Guided Percutaneous Needle Electrolysis (PNE)

PNE was administered in three sessions (on days 1, 7 and 14) under ultrasound guidance, following the protocol developed by the MVClinic Institute [38] for the treatment of tendinopathies. Patients were placed supine with approximately 30° of knee flexion, supported by a pillow. Prior to the intervention, the treatment area was sterilized with 2% chlorhexidine, and new needles and ultrasound probe covers were used for each session. Based on the baseline ultrasound evaluation, the intervention protocol was selected based on the lesion’s location and characteristics. When the insertional area of the patellar tendon at the inferior pole of the patella was affected, a longitudinal (long-axis) view of the patellar tendon was used and the intervention consisted of a long-axis approach from distal to proximal, with the needle oriented at 45°, applying a galvanic current of 3 mA for three seconds to the previously identified ultrasonographic lesion area. After each pulse, the needle was repositioned slightly in three dimensions to target different regions of the tissue. This process was repeated three times using the same parameters. However, if a loss of continuity of the deep interface between the patellar tendon and Hoffa’s fat pad was detected during the baseline ultrasound evaluation, a transverse (short-axis) view of the tendon was performed using a medial-to-lateral approach with a 15° needle orientation. This consisted of two phases: three needle advances in the forward (anterograde) direction, followed by a final application in the backward (retrograde) direction. A galvanic current of 1.5 mA was applied for three seconds in each phase (1.5:3:4) (Intensity [mA]: Time [seconds]: Impacts [number applications]) [38].

#### 2.5.3. Ultrasound-Guided Percutaneous Neuromodulation (PNM)

PNM intervention was performed on days 1, 7, and 14 of the treatment period, immediately after PNE was applied. Patients were also placed supine with approximately 30 degrees of knee flexion, supported by a pillow. Under ultrasound guidance, the femoral nerve was targeted immediately distal to the inguinal ligament. A surface electrode patch was placed over the patellar tendon to complete the electrical circuit. Stimulation was delivered at 2 Hz with a pulse width of 250 μs and an intensity adjusted to achieve a comfortable muscle contraction threshold without inducing pain. Stimulation was maintained for 25 min, in accordance with the protocol established by the MVClinic Institute [17].

### 2.6. Statistical Analysis

All statistical analyses were performed using R software, version 4.5.2. The significance level was set at *p* < 0.05. Baseline characteristics were summarized by the treatment group. Continuous variables were described using the mean and standard deviation (SD), as well as the median and interquartile range (IQR), whereas categorical variables were described using frequencies and percentages. To visually explore the distribution and evolution of continuous outcomes over time, violin plots were combined with box plots, stratified by treatment group. These plots display the full distribution of the data at each time point, including density, central tendency, and dispersion. The individual trajectories of each participant were also overlaid as line plots, which enabled visualization of changes within subjects over time and facilitated identification of variability in treatment response.

Prior to the primary analyses, baseline characteristics were compared between groups to identify clinically relevant imbalances that could potentially influence treatment response. Given the exploratory nature of these comparisons and current recommendations discouraging inferential testing of baseline differences in randomized studies [22], these analyses used only to inform sensitivity analyses rather than to guide the primary modeling strategy. Continuous variables were compared using the Mann–Whitney U test, given the limited sample size, and categorical variables were compared using Fisher’s exact test, given the low expected frequency across the different levels.

The primary outcome of pain intensity, as measured by the VAS, was analyzed using a linear mixed-effect model. This model included fixed effects for treatment group and post-treatment time (Days 7, 14, and 21), as well as their interaction (Group × Time). Baseline VAS (Day 1) was included as a covariate to adjust for initial differences. Model results were summarized using Type III sum-of-squares analysis. Adjusted estimated marginal means were obtained for each treatment group at each time point. Post hoc, between-group, pairwise comparisons were then performed and the results reported as mean differences with 95% confidence intervals and standardized effect sizes (Cohen’s d). This post hoc analysis was restricted to between-group comparisons; no within-group post hoc analyses were performed. This approach was consistent with the study design and primary objective of determining whether adding PNM (experimental group) to treatment comprising EE and PNE (active control group) provided additional benefit. Accordingly, the analysis focused on between-group differences rather than within-group changes from baseline.

The other continuous outcomes, VISA-P and SF-36, which were assessed at baseline and on Day 21, were analyzed using analysis of covariance (ANCOVA). The post-treatment value was used as the dependent variable, the treatment group was used as the fixed factor, and the baseline value of the same outcome was used as the covariate. Adjusted estimated marginal means were calculated for each group, and between-group differences were reported with 95% confidence intervals and standardized effect sizes. As previously stated, post hoc analyses for these variables were conducted only to assess between-group differences. Model assumptions were assessed graphically and analytically, including assessment of residual normality, homoscedasticity and influential observations.

To assess the potential influence of baseline imbalances on treatment outcomes, sensitivity analyses were conducted by progressively incorporating the variables identified in the initial comparison as additional covariates into the main models both individually and in combination. Given the limited sample size, inclusion of multiple covariates substantially reduces degrees of freedom and increases the risk of model overfitting and unstable parameter estimation. Therefore, the primary analyses were intentionally based on parsimonious models adjusted only for baseline outcome values, and the sensitivity analyses were performed exclusively to evaluate the robustness of the treatment effect estimates to potential baseline imbalances.

Due to their nature and the limited sample size, ordinal outcomes (including the Pistol Squat test and ultrasound imaging variables such as focal hypoechoic area, neovascularization, focal hyperechoic area, deep interface continuity and tendon thickness) were analyzed using non-parametric methods. The change from Day 1 to Day 21 was calculated for each participant and compared between groups using the Mann–Whitney U test. Additionally, the results were categorized as improved, no change or worsened and summarized as frequencies and percentages for each treatment group. For binary outcomes, between-group comparisons were performed using Fisher’s exact test.

## 3. Results

### 3.1. Participant Flow and Baseline Sociodemographic Data

**Figure 2:**
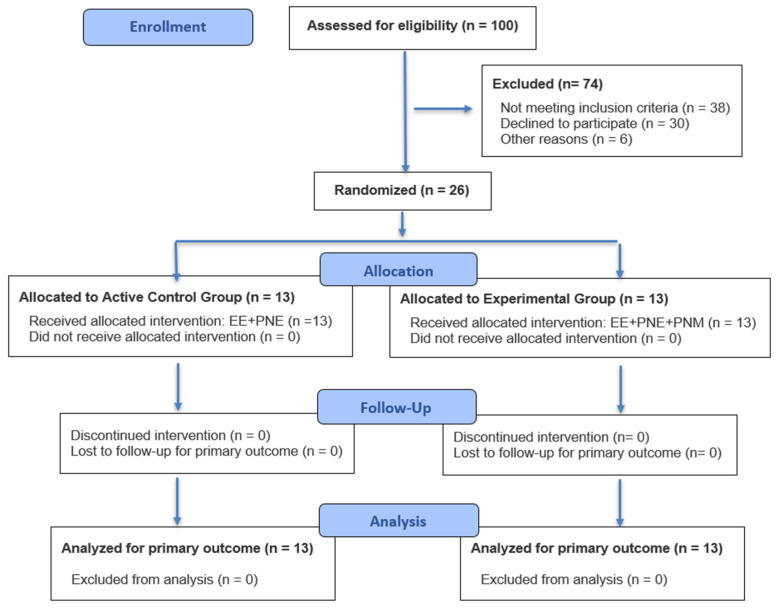
Participants flow chart.

The initial questionnaire, which was used as a preliminary screening tool, was completed by 100 participants between February and April 2025. Of these, 74 were excluded for not meeting the eligibility criteria. Following individual contact, the remaining eligible participants were screened with the rest of eligibility criteria and 26 participants who met all the eligibility criteria were included in this study. Participants were randomly allocated in a 1:1 ratio to two groups: the active control group (EE+PNE) and the experimental group (EE+PNE+PNM) (Figure 2). Baseline sociodemographic characteristics of the sample are presented in Table 1.

### 3.2. Clinical Outcomes

To evaluate the robustness of the primary findings against potential confounding, sensitivity analyses were conducted for all primary outcome models. These analyses progressively incorporated sex and body mass index, which exhibited baseline imbalances between groups (see Table 1), as well as symptom chronicity, selected due to its clinical relevance and potential influence on treatment response [39,40].

#### 3.2.1. Pain Intensity (VAS)

**Figure 3:**
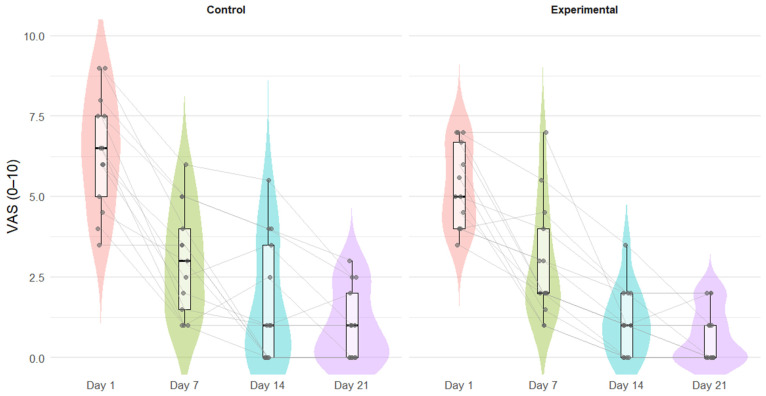
Pain intensity (VAS) over time by treatment group. Violin plots, box plots, individual data points and trend lines for each subject, by treatment group, are presented.

Significant changes in pain intensity were observed over time (time effect: *p* < 0.001), with a progressive reduction seen in both groups from day 1 to day 21. However, no significant differences were found between the groups (group effect: *p* = 0.844) or in the group × time interaction (*p* = 0.561). Post hoc analyses revealed no significant differences between groups at any point during follow-up (days 7, 14 and 21), with small, non-significant effect sizes (see Table 2). Visual inspection supported the assumptions of normality and homoscedasticity. Figure 3 shows the violin plots for the data from each group at each time point.

Sensitivity analyses incorporating sex, body mass index, and symptom chronicity as additional covariates, both individually and in combination, yielded results consistent with the primary model. Neither the group effect nor the group × time interaction reached statistical significance in any adjusted model, and effect estimates remained stable across analyses.

#### 3.2.2. Functional Disability (VISA-P) and Quality of Life (SF-36)

**Figure 4:**
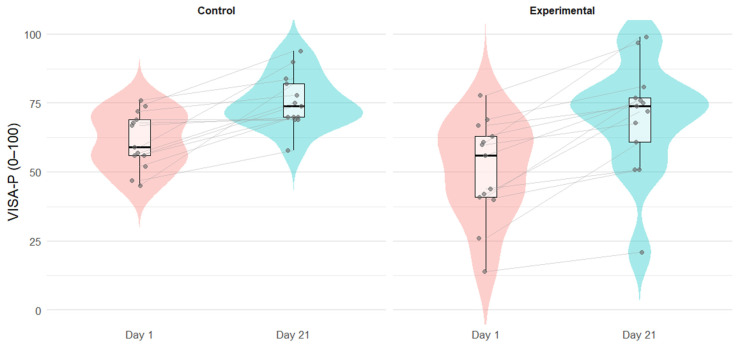
Functional disability (VISA-P) over time by treatment group. Violin plots, box plots, individual data points and trend lines for each subject, by treatment group, are presented.

**Figure 5:**
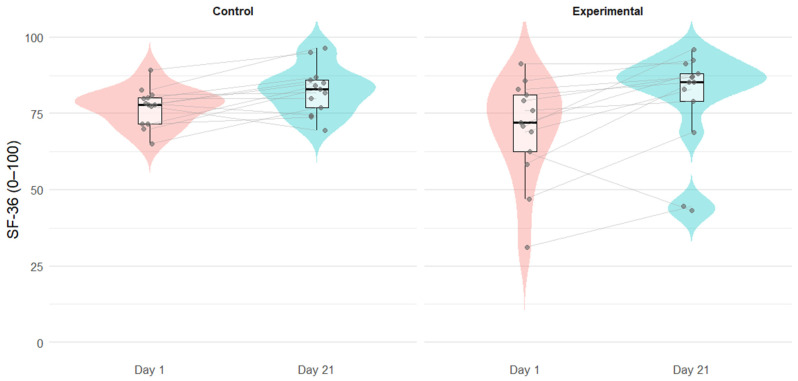
Quality of life (SF-36) over time by treatment group. Violin plots, box plots, individual data points, and trend lines for each subject, by treatment group, are presented.

Pre–post within-group changes were observed for both variables from day 1 to day 21 in both groups (Figure 4 and Figure 5). However, post-treatment between-group analyses, adjusted for baseline values using ANCOVA, revealed no significant differences in functional disability (*p* = 0.181) or quality of life (*p* = 0.400). Post hoc analyses confirmed the absence of significant differences between the groups, with small effect sizes (see Table 2). Visual inspection supported the assumptions of normality and homoscedasticity in both models.

In addition, the inclusion of sex, body mass index, and symptom chronicity as additional covariates did not materially alter the group effect estimates or statistical significance compared with the primary ANCOVA models.

#### 3.2.3. Functional Capacity (Pistol Squat Test)

According to the descriptive data, improvements in functional capacity were observed in both groups at the end of the follow-up period. In the active control group, 92.3% of participants showed improvement, whereas in the experimental group, all participants did. However, no statistically significant differences were found between the two groups (*p* = 0.716; see Table 3).

#### 3.2.4. Ultrasound Imaging Outcomes

Descriptive data showed that none of the participants experienced any deterioration in the assessed variables. Furthermore, a consistent trend was observed across all ultrasound variables: participants presenting with abnormalities at the start of this study showed improvement following the corresponding intervention. Notably, the most relevant changes were observed in cases that were initially classified as “large” alterations. Specifically, focal hypoechoic areas and tendon thickening categorized as large were reduced in all cases, and neovascularization graded as large was almost completely reduced across participants. A high proportion of participants showed no change during the follow-up period, which can be attributed to their baseline values already being within normal limits. No statistically significant differences between groups were found for any of the ultrasound variables (see Table 3).

## 4. Discussion

Although there is currently no evidence regarding the isolated effect of PNM in patients with PT, recent literature suggests that PNM may have beneficial effects on pain, range of motion, functionality and quality of life in patients with different knee pathologies. Of all the available evidence, only one study employed a methodology comparable to that of the present study: an ultrasound-guided intervention without any implanted devices in a population experiencing unilateral anterior knee pain due to chondropathy, meniscopathy or internal meniscus arthroscopy [41]. In contrast, most studies investigating PNM for knee conditions focus on medical implantable devices that remain in the patient’s body for about 60 days, with stimulation delivered via remote control; this approach is not directly comparable to the ultrasound-guided percutaneous intervention used in the present study. This type of PNM has been studied in patients undergoing total knee replacement [42,43] and in individuals with osteoarthritis [44,45].

Evidence regarding isolated PNM also remains limited when the scope is extended to other tendinopathies, with only one pilot study performed with participants with chronic lateral epicondylalgia, which reported positive effects of ultrasound-guided PNM on pain, functionality, neural excitability, and morphology [46]. Regarding the combined use of PNM with other interventions, a recent study similar to ours investigated patients with supraspinatus tendinopathy [47]. In that study, the authors combined PNM, PNE and EE, and compared this with conventional physiotherapy (ultrasound therapy, transcutaneous electrical nerve stimulation, and EE). They found that the PNM+PNE+EE group showed statistically significant improvements in pain, mobility and functionality throughout the follow-up period (from baseline up to 24 weeks). In addition, this group showed significantly greater improvements compared to the conventional physiotherapy across nearly all these variables at the different follow-up time points (post-treatment, 12, and 24 weeks).

As previously mentioned, the evidence supporting the use of ultrasound-guided PNM in the treatment of tendinopathy remains limited. Furthermore, studies involving PNM in knee pathology either lack a control group or include placebo comparators, and typically administer PNM as an isolated intervention. Consequently, it is unclear whether adding PNM to an evidence-based treatment provides any additional benefit. Notably, the aforementioned study [47] on supraspinatus tendinopathy combined PNM with an active treatment (PNE+EE) but did not include a direct comparison with a group receiving the same intervention without PNM. For this reason, the present study aimed to determine whether adding PNM to a combined EE+PNE intervention provides additional benefit in patients with PT, rather than evaluating the isolated effect of PNM.

The results of the present study showed that adding PNM to PNE+EE treatment (active control group) did not result in any statistically significant additional benefits in the short term (21-day follow-up) after three treatment sessions (days 1, 7 and 14). These findings may be explained by the strong evidence supporting the effectiveness of EE as a standalone intervention or when combined with sham therapies [15,48,49], as well as when combined with PNE [50,51] in patients with PT. This suggests that these interventions may already be approaching a therapeutic ceiling effect, leaving limited room for additional benefit from PNM. Future studies could explore the opposite hypothesis, whereby both groups receive PNM and only one group receives PNE in addition, to determine whether PNM itself may reach a similar therapeutic ceiling. However, caution is warranted when interpreting these findings, as the lack of statistically significant between-group differences only indicates that no additional benefit of EE+PNE+PNM protocol was detected within the context of the present study and should not be interpreted as evidence of equivalence, non-inferiority, or a definitive existence of therapeutic ceiling effect.

As a speculative explanation, the therapeutic ceiling hypothesis could be supported by robust evidence indicating that the most important factor in successfully managing tendinopathy is progressively controlling and increasing the mechanical load applied to the tendon. This is typically achieved through exercise programs based on EE [11,52,53]. The effectiveness of progressive exercise, and specifically EE, is clearly demonstrated in two studies by López-Royo et al. (2021 and 2024) [15,49], in which isolated EE (combined with sham dry needling) was found to be as effective as EE+PNE. These findings emphasize the significant clinical impact of therapeutic exercise, particularly EE, in PT, which could limit the added value of percutaneous interventions such as PNE or PNM.

Conversely, a recent study by Caballero-López et al. (2025) [54] found that incorporating PNM targeting the femoral nerve into a rehabilitation program provided additional benefits for patients undergoing anterior cruciate ligament (ACL) surgery. These benefits included short-term pain reduction (up to four weeks), improvements in range of motion and pressure pain threshold (up to 12 weeks), and short-term increases in quadriceps isometric strength immediately after the intervention. A key difference between the two studies lies in the population studied. The former study included patients undergoing ACL surgery, a condition primarily affecting passive joint structures, whereas the latter focused on PT, which involves an active structure. This suggests that exercise-based rehabilitation may be less effective in conditions that affect passive structures, thereby allowing for greater potential for additional benefits from PNM. Furthermore, the control group in the aforementioned study received multimodal rehabilitation (mobility, neuromuscular electrical stimulation, manual therapy, and exercise), whereas the active control group in the present study received three sessions of PNE in addition to EE, which may have reduced the likelihood of detecting additional benefits from PNM [54].

Focusing on the variables in our study, both groups showed significant improvements over time in pain intensity (VAS), with no group demonstrating superiority over the other. These improvements in pain are consistent with previous literature on PNE in tendinopathies [20,51].

Similarly, quality of life (SF-36) improved in both groups, with no statistically significant difference between them. However, while these are not specific PT investigations, studies of populations undergoing partial and total knee arthroplasty reported that the minimum clinically important differences (MCIDs) for the SF-36 questionnaire were 11.3 and 4.6 points, respectively [55,56]. In the present study, only the experimental group (EE+PNE+PNM) exceeded these two thresholds (13.4 points), while the active control group (EE+PNE) showed a smaller improvement (5 points). At the individual level, 46.2% of participants in the experimental group exceeded the higher threshold (11.3 points) and 61.5% exceeded the lower threshold (4.6 points), compared with 30.8% and 61.5%, respectively, in the active control group. These findings suggest that adding PNM to the intervention may help to achieve clinical significance in a subset of patients who may particularly benefit from this approach.

Regarding functional disability (VISA-P), no statistically significant differences were observed between the groups. In this case, a change of 13–14 points has been established as the MCID, corresponding to a 98% probability of clinical significance [57]. Subsequent studies have also adopted this threshold of 13–14 points [58,59,60,61]. The experimental group (EE+PNE+PNM) showed a greater mean improvement than the active control group (EE+PNE): 18.61 and 14.23 points, respectively. However, at an individual level, 46.2% of participants in the experimental group exceeded the MCID threshold compared to 53.8% in the active control group, indicating minimal differences in terms of clinically significant improvement. Regarding functional capacity, both groups demonstrated improvements in pistol squat performance, with more than 90% of participants improving from baseline, consistent with previous studies [50]. Although recent data [62,63] suggest that PNM may directly affect functional outcomes, the assessment methods used in this study (a self-reported questionnaire and an ordinal functional measure) may have limited our ability to capture its full impact.

When interpreting these findings, it is imperative to distinguish between statistical significance and clinical relevance. Statistical significance, commonly evaluated through *p*-values, does not constitute a measure of clinical importance nor prove that a result is real or plausible; rather, it only reflects the probability of obtaining a result equal to or more extreme than the observed data, assuming that the null hypothesis is true and all statistical assumptions are met. Clinical relevance is often estimated using the MCID, which represents the smallest change perceived as meaningful by patients. Nevertheless, MCID values should be considered as guidance rather than strict categorical thresholds for clinical decision-making, and their interpretation should always be complemented by the direct individual patient’s subjective perception of improvement.

Finally, ultrasound imaging outcomes also indicated improvements, primarily reductions in the size of hypoechoic areas within the tendon, neovascularization, and tendon thickness in both groups. These results are consistent with those of previous ultrasound studies of PT [64] and other tendinopathies [65]. However, it is important to note that ultrasound improvements do not always correlate with clinical improvements and should therefore be interpreted with caution. The literature indicates that structural abnormalities, such as hypoechoic areas or neovascularization, are commonly observed in asymptomatic athletes who continue to perform at a high level without symptoms [66]. Conversely, significant clinical changes in pain and function often precede or occur independently of noticeable structural normalization on imaging [67], as it was observed in a recent randomized controlled trial that also administered PNM for PT [15]. This clinical-structural dissociation underscores that imaging findings should be utilized to complement, rather than dictate, clinical reasoning and prognosis [15]. Regarding tendon thickness, a recent study found that the difference in thickness between a tendon with PT and a “healthy” tendon on the opposite side was 2.5 mm in the proximal part and 0.5 mm in the distal part [68]. In the present study, most patients presented with proximal involvement, and all those classified as having large tendon thickening (>1 mm) at baseline improved to small (≤1 mm) or absent tendon thickening, regardless of group allocation.

Overall, the findings from this study support the use of combined invasive physiotherapy techniques (PNE, with or without PNM) alongside exercise (EE) as an effective PT treatment strategy. However, they reinforce the idea that progressive mechanical loading of the tendon remains the key therapeutic factor, meaning that the decision to use these invasive physiotherapy techniques depends on the professional’s subjective judgment, given the need to provide personalized treatments based on clinical reasoning. Notably, the relatively rapid changes observed in ultrasound imaging outcomes between baseline and day 21 may suggest an additional contribution of PNE to early structural tendon adaptation. Given the typically slower timeline reported for structural changes induced by exercise alone, it is plausible that these early improvements would not have been observed to the same extent with exercise as a standalone intervention.

Several limitations of this study should be acknowledged. First, although previous studies evaluating PNM in other tendinopathies have successfully reported positive clinical and functional outcomes after short-term follow-ups of 4 weeks [46,47], the 21-day follow-up employed in the present trial is insufficient to adequately assess structural tendon remodeling, which depends on mechanotransduction processes and the inherently slow turnover of collagen. Likewise, the evaluation of sustained treatment effects in chronic tendinopathies generally requires follow-up periods extending over several months. Consequently, the findings of the present study are limited to short-term adaptations and should be interpreted within the context of biological constraint. In addition, most outcome variables were assessed only at baseline and at the final follow-up, precluding analysis of intermediate changes and the temporal evolution of treatment effects. Future trials must incorporate both additional assessment time points and extended follow-up periods (e.g., 6 to 12 months) to determine the progression of clinical, functional, and structural adaptations over time.

Regarding internal validity, despite randomization, baseline imbalances were observed between groups in sex distribution and body mass index, while symptom chronicity and body weight also showed noticeable numerical differences, though they did not reach statistical significance. Sensitivity analyses incorporating these variables as additional covariates yielded consistent treatment-effect estimates across all model specifications, suggesting that the primary findings were robust to these imbalances. Nevertheless, residual confounding cannot be completely excluded, particularly given the limited sample size. Furthermore, the absence of a sham-stimulation control group or an exercise-only arm limits our ability to fully isolate the specific therapeutic contribution of PNM. Future studies with larger samples and additional comparator groups are therefore warranted to strengthen causal inference and to further explore the potential moderating role of clinical and sociodemographic variables on treatment response.

Some limitations also relate to the measurement tools used in this study. Ultrasound imaging variables were primarily evaluated using descriptive and qualitative ordinal scales rather than continuous metrics. Although this approach offers high clinical reproducibility and facilitates interpretation in routine practice, it may reduce sensitivity to detect subtle structural tendon changes. Similarly, functional outcomes were assessed using a self-report questionnaire (VISA-P) and an ordinal functional measure (performance in the pistol squat). As a result, subtle neuromuscular adaptations following PNM may not have been captured by subjective measures. Future studies therefore consider incorporating more sensitive and objective assessment methods, including quantitative imaging tools, such as ultrasound tissue characterization or texture analysis, as well as isokinetic dynamometry and electromyography, to better characterize tissue remodeling and functional and neuromuscular adaptations associated with PNM treatment.

Finally, although comparisons were made with established MCID thresholds to assess the clinical significance of the observed changes, these should be interpreted with caution. MCID values only provide an estimate of significant change at the group level and may not accurately reflect how each patient perceives improvement. Future studies should therefore consider incorporating patient-reported global change scales to more accurately reflect individual perceptions of treatment efficacy. In addition, the sample consisted of physically active individuals with predominantly chronic proximal PT, a relatively specific population that may limit the generalizability of the findings to other clinical presentations. Further research should therefore investigate the influence of different clinical and sociodemographic factors, including tendon location, symptom duration, severity level, and patient profiles, to determine the broader applicability of these findings.

## 5. Conclusions

Adding ultrasound-guided percutaneous neuromodulation (PNM) to a treatment based on percutaneous needle electrolysis and eccentric exercises (PNE+EE) did not result in any statistically significant additional benefits in the short term for patients with patellar tendinopathy (PT) when analyzed based on any of the following variables: pain intensity, functionality, quality of life, and ultrasound imaging parameters. Although the absence of between-group differences may suggest that the addition of PNM did not provide further benefit beyond the effects achieved with the PNE+EE protocol, this interpretation should be considered exploratory. Future adequately powered studies, ideally employing non-inferiority or equivalence designs, are needed to determine whether a ceiling effect may explain the observed findings.

## Figures and Tables

**Table 1:** Baseline sociodemographic data.

Variable		Control Group: EE+PNE (n = 13)	Experimental Group: EE+PNE+PNM (n = 13)	Between-Group Comparison (*p*-Value)
Gender	Male	12 (92.3%)	6 (46.2%)	0.030 *
	Female	1 (7.7%)	7 (53.8%)	
Age (years)		31.15 ± 9.09 30 (25, 38)	35.69 ± 9.93 36 (28, 41)	0.400
Weight (kg)		76.25 ± 9.93 76 (74, 81)	68.23 ± 13.16 73 (55, 75)	0.068
Height (cm)		176.77 ± 5.49 177 (173, 180)	173.38 ± 7.43 172 (170, 180)	0.300
BMI (Weight/Height(m)^2^)		24.36 ± 2.54 25.65 (22.69, 25.85)	22.57 ± 3.45 22.28 (20.70, 24.07)	0.044 *
Weekly training (days per week)		4.62 ± 1.61 5 (3, 6)	4.69 ± 2.02 4 (3, 7)	0.900
Affected side	Right	7 (53.8%)	5 (38.5%)	0.700
	Left	6 (46.2%)	8 (61.5%)	
Chronicity (months)		29.62 ± 35.23 18 (7, 24)	64.38 ± 73.20 36 (18, 60)	0.110
Previous treatment	None	9 (69.2%)	7 (53.8%)	0.800
	PNE	3 (23.1%)	3 (23.1%)	
	DN	0 (0%)	2 (15.4%)	
	PRP	1 (7.7%)	1 (7.7%)	
Intervention area in the current study	A1	12 (92.3%)	9 (69.2%)	0.400
	A2	0 (0%)	2 (15.4%)	
	A3	0 (0%)	0 (0%)	
	A4	0 (0%)	0 (0%)	
	A5	1 (7.7%)	2 (15.4%)	

Data are presented as mean ± standard deviation and median (IQR) for quantitative variables, and as frequencies (percentages) for qualitative variables. A1: Proximal insertion of the deep fibers of the patellar tendon into the patella; A2: Deep interface between the patellar tendon and Hoffa’s fat pad; A3: Body of the tendon; A4: Distal insertion of the deep fibers of the patellar tendon into the tibia; A5: Distal insertion of the superficial fibers of the patellar tendon into the tibia; DN: dry needling; EE: eccentric exercises; PNE: percutaneous needle electrolysis; PNM: percutaneous neuromodulation; PRP: platelet-rich plasma. * Statistically significant differences: *p* < 0.05.

**Table 2:** Descriptive and statistical results for clinical (quantitative) outcomes.

	Active Control Group: EE+PNE (n = 13)				Experimental Group: EE+PNE+PNM (n = 13)				Model Results: F-Value (*p*-Value)			Between-Groups Post Hoc Comparisons: Mean Difference [CI95%] (*p*-Value) Effect Size [CI95%]		
	Baseline (Day 1)	Day 7	Day 14	Day 21	Baseline (Day 1)	Day 7	Day 14	Day 21	Group Effect	Time Effect	Group × Time Interaction	Day 7	Day 14	Day 21
Pain intensity (VAS)	6.38 ± 1.79 6.50 (5, 7.50)	3.00 ± 1.68 3 (1.50, 4)	1.73 ± 1.93 1 (0, 3.50)	1.00 ± 1.14 1 (0, 2.00)	5.33 ± 1.30 5 (4, 6.70)	2.96 ± 1.82 2 (2, 4)	1.12 ± 1.04 1 (0, 2)	0.69 ± 0.85 0 (0, 1)	0.04 (0.844) ^A^	34.19 (<0.001) *^,A^	0.58 (0.561) ^A^	0.37 [−0.74–1.49] (0.504) ^C^ 0.39 [−0.77–1.55] ^D^	−0.20 [−1.32–0.91] (0.715) ^C^ −0.21 [−1.37–0.95] ^D^	0.10 [−1.01–1.22] (0.852) ^C^ 0.11 [−1.05–1.27] ^D^
Functional disability (VISA-P)	61.38 ± 10.27 59 (56, 69)	-	-	75.62 ± 9.82 74 (70, 82)	50.85 ± 18.27 56 (41, 63)	-	-	69.46 ± 20.46 74 (61, 77)	1.90 (0.181) ^B^	-	-	-	-	2.06 [−7.79–11.9] (0.669) ^C^ 0.18 [0.69–1.05] ^D^
Quality of life (SF-36)	77.17 ± 6.27 78 (71.67, 80.10)	-	-	82.56 ± 7.82 83 (77, 86)	69.85 ± 16.73 72 (62.64, 81.25)	-	-	79.34 ± 17.07 85.40 (79, 88)	0.734 (0.400) ^B^	-	-	-	-	2.22 [−5.90–10.30] (0.577) ^C^ 0.23 [−0.62–1.08] ^D^

Descriptive data are presented as mean ± standard deviation and median (IQR). Day 7 and day 14 correspond to assessments of pain intensity performed prior to the second and third treatment sessions, respectively, of the allocated percutaneous intervention. EE: eccentric exercises; IQR: interquartile range; PNE: percutaneous needle electrolysis; PNM: percutaneous neuromodulation; SF-36: short-form 36 questionnaire; VAS: visual analog scale; VISA-P: Victorian Institute of Sport Assessment-Patella questionnaire. *: statistically significant difference; ^A^: linear mixed-effect model with baseline VAS included as a covariate; ^B^: ANCOVA model; ^C^: adjusted estimated marginal means; ^D^: Cohen’s d.

**Table 3:** Descriptive and statistical results for qualitative outcomes.

		Active Control Group: EE+PNE (n = 13)		Experimental Group: EE+PNE+PNM (n = 13)		Descriptive Pre–Post Change Frequency (%)		Between-Groups Comparisons (*p*-Value)
		Baseline (Day 1)	Day 21	Baseline (Day 1)	Day 21	Active Control Group	Experimental Group	
Functional capacity (Pistol squat test)	No pain	1 (7.7%)	6 (46.2%)	0 (0%)	6 (46.2%)	Improved: 12 (92.3%) No change: 1 (7.7%) Worsened: 0 (0%)	Improved: 13 (100%) No change: 0 (0%) Worsened: 0 (0%)	(0.716) ^A^
	Mild pain	0 (0%)	7 (53.8%)	0 (0%)	6 (46.2%)			
	Pain	6 (46.2%)	0 (0%)	6 (46.2%)	1 (7.7%)			
	Inability	6 (46.2%)	0 (0%)	7 (53.8%)	0 (0%)			
Focal hyperechoic area	Absent	13 (100.0%)	13 (100.0%)	12 (92.3%)	13 (100.0%)	Improved: 0 (0%) No change: 13 (100%) Worsened: 0 (0%)	Improved: 1 (7.7%) No change: 12 (92.3%) Worsened: 0 (0%)	(0.356) ^A^
	Small	0 (0%)	0 (0%)	0 (0%)	0 (0%)			
	Large	0 (0%)	0 (0%)	1 (7.7%)	0 (0%)			
Focal hypoechoic area	Absent	1 (7.7%)	2 (15.4%)	3 (23.1%)	5 (38.5%)	Improved: 12 (92.3%) No change: 1 (7.7%) Worsened: 0 (0%)	Improved: 10 (76.9%) No change: 3 (23.1%) Worsened: 0 (0%)	(0.717) ^A^
	Small	0 (0%)	11 (84.6%)	0 (0%)	8 (61.5%)			
	Large	12 (92.3%)	0 (0%)	10 (76.9%)	0 (0%)			
Neovascularization	Absent	8 (61.5%)	10 (76.9%)	8 (61.5%)	9 (69.2%)	Improved: 4 (30.8%) No change: 9 (69.2%) Worsened: 0 (0%)	Improved: 5 (38.5%) No change: 8 (61.5%) Worsened: 0 (0%)	(0.855) ^A^
	Small	0 (0%)	2 (15.4%)	0 (0%)	4 (30.8%)			
	Large	5 (38.5%)	0 (0%)	5 (38.5%)	0 (0%)			
Thickening of the tendon	Absent	5 (38.5%)	9 (69.2%)	6 (46.2%)	7 (53.8%)	Improved: 8 (61.5%) No change: 5 (38.5%) Worsened: 0 (0%)	Improved: 7 (53.8%) No change: 6 (46.2%) Worsened: 0 (0%)	(0.377) ^A^
	Small	0 (0%)	4 (30.8%)	0 (0%)	6 (46.2%)			
	Large	8 (61.5%)	1 (7.7%)	7 (53.8%)	0 (0%)			
Continuity of the deep interface	Preserved	13 (100.0%)	13 (100.0%)	11 (84.6%)	13 (100.0%)	Improved: 0 (0%) No change: 13 (100%) Worsened: 0 (0%)	Improved: 2 (15.4%) No change: 11 (84.6%) Worsened: 0 (0%)	(0.480) ^B^
	Not preserved	0 (0%)	0 (0%)	2 (15.4%)	0 (0%)			

Descriptive data are presented as frequencies (percentages). EE: eccentric exercises; PNE: percutaneous needle electrolysis; PNM: percutaneous neuromodulation. ^A^: Mann–Whitney U test; ^B^: Fisher’s exact test.

## Data Availability

The data presented in this study are available on request from the corresponding author. The data are not publicly available due to privacy restriction.

## Supplementary Materials

The following supporting information can be downloaded at: https://www.mdpi.com/article/10.3390/healthcare14142092/s1. Supplementary File S1: CONSORT 2025 checklist of information to include when reporting a randomised trial.

## Abbreviations

The following abbreviations are used in this manuscript:

ACL	Anterior Cruciate Ligament
ANCOVA	Analysis of Covariance
EE	Eccentric Exercise
ICC	Intraclass Correlation Coefficient
IQR	Interquartile Range
MCID	Minimum Clinically Important Difference
PNE	Percutaneous Needle Electrolysis
PNM	Percutaneous Neuromodulation
PT	Patellar Tendinopathy
SD	Standard Deviation
VAS	Visual Analogue Scale
VISA-P	Victorian Institute of Sport Assessment–Patella questionnaire
